# Carboxymethyl Hyaluronan-Stabilized Nanoparticles for Anticancer Drug Delivery

**DOI:** 10.1155/2015/249573

**Published:** 2015-09-10

**Authors:** Jessica L. Woodman, Min Sung Suh, Jianxing Zhang, Yuvabharath Kondaveeti, Diane J. Burgess, Bruce A. White, Glenn D. Prestwich, Liisa T. Kuhn

**Affiliations:** ^1^Department of Materials Science and Engineering, University of Connecticut, Storrs, CT 06269, USA; ^2^Department of Reconstructive Sciences, UConn Health, Farmington, CT 06030, USA; ^3^Department of Pharmaceutical Sciences, University of Connecticut, Storrs, CT 06269, USA; ^4^Department of Medicinal Chemistry, University of Utah, Salt Lake City, UT 84108, USA; ^5^Department of Cell Biology, UConn Health, Farmington, CT 06030, USA

## Abstract

Carboxymethyl hyaluronic acid (CMHA) is a semisynthetic derivative of HA that is recognized by HA binding proteins but contains an additional carboxylic acid on some of the 6-hydroxyl groups of the N-acetyl glucosamine sugar units. These studies tested the ability of CMHA to stabilize the formation of calcium phosphate nanoparticles and evaluated their potential to target therapy resistant, CD44^+^/CD24^−/low^ human breast cancer cells (BT-474_EMT_). CMHA stabilized particles (nCaP^CMHA^) were loaded with the chemotherapy drug *cis*-diamminedichloroplatinum(II) (CDDP) to form nCaP^CMHA^CDDP. nCaP^CMHA^CDDP was determined to be poorly crystalline hydroxyapatite, 200 nm in diameter with a −43 mV zeta potential. nCaP^CMHA^CDDP exhibited a two-day burst release of CDDP that tapered resulting in 86% release by 7 days. Surface plasmon resonance showed that nCaP^CMHA^CDDP binds to CD44, but less effectively than CMHA or hyaluronan. nCaP^CMHA-AF488^ was taken up by CD44^+^/CD24^−^ BT-474_EMT_ breast cancer cells within 18 hours. nCaP^CMHA^CDDP was as cytotoxic as free CDDP against the BT-474_EMT_ cells. Subcutaneous BT-474_EMT_ tumors were more reproducibly inhibited by a near tumor dose of 2.8 mg/kg CDDP than a 7 mg/kg dose nCaP^CMHA^CDDP. This was likely due to a lack of distribution of nCaP^CMHA^CDDP throughout the dense tumor tissue that limited drug diffusion.

## 1. Introduction

In the United States, over 1.6 million people were newly diagnosed with cancer in 2013 and 13.7 million people were battling cancer or were in remission [1]. Approximately 1 in 5 breast cancer survivors will have a recurrence within 10 years of adjuvant therapy [2]. While chemotherapies generally target rapidly dividing cells, relatively dormant cells exist within tumors which are resistant to chemotherapy [3–6]. Therapy-resistant breast cancer cells have a common phenotype of CD44^+^/CD24^−/low^, in which CD44 is a transmembrane hyaluronan (HA) receptor. HA is a major glycosaminoglycan component of the extracellular matrix [7]. The expression of CD44 has been linked to cancer progression via metastases and drug resistance [8]. The presence of CD44^+^ cells in patients with triple negative breast cancer (TNBC) is an indicator of poor outcomes and is linked with recurrence [9]. TNBC patients have limited treatment options, because their cancer does not present with hormone receptors that are effectively targeted for treatment [10]. These patients could greatly benefit from a localized high dose treatment that could reduce the tumor size prior to surgical resection, thereby reducing the incidence of recurrence. Two histological studies examining breast cancer patient tumor samples prior to and after primary systemic chemotherapy evidenced an increase in therapy-resistant CD44^+^/CD24^−/low^ cells after treatment [5, 11]. The objective of this work was to determine whether calcium phosphate nanoparticles stabilized with a chemically modified HA could effectively target and kill therapy-resistant human TNBC cells with the CD44^+^/CD24^low^ phenotype.

**Figure 1:**
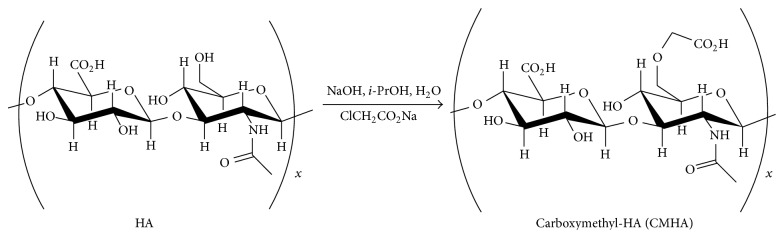
Chemical modification of hyaluronan (HA) to carboxymethyl hyaluronan (CMHA). HA was chemically modified to produce CMHA in a basic solution which modifies approximately 15–20% of the 6′-OH groups of the N-acetylglucosamine residues. This process reduces the molecular weight and introduces additional carboxyl groups for stabilization of nCaP.

Utilizing HA as a targeting moiety to deliver chemotherapeutics to cancer cells has been an ongoing area of research. One approach has been to chemically modify HA to allow attachment of carboxyl-containing drugs [12], and an HA-Paclitaxel prodrug bioconjugate has been prepared that showed selective toxicity against cancer cells overexpressing CD44 [13, 14]. Drug carrier systems modified with HA have been shown to enter the cell via CD44 mediated endocytosis [15]. Chen et al. showed mesoporous silica nanoparticles targeted with HA entered cells expressing CD44 [16]. In this study targeted calcium phosphate nanoparticles (nCaP) were synthesized as the drug carrier. Calcium phosphate is an excellent biomaterial because it is biocompatible, resorbable, and nonimmunogenic [17–19]. CaP synthesized via wet precipitation will form microcrystals, instead of nanoparticles, if crystallization and agglomeration are not halted with a stabilizer. Limiting crystal growth and agglomeration with a stabilizer is important to improve injectability of colloidal suspensions of CaP used for drug delivery. In the present studies, chemically modified HA was evaluated as a dual stabilizer/targeting ligand for nCaP. The chemically modified HA had additional carboxylate groups installed on a predetermined fraction of the N-acetylglucosamine units at the 6-hydroxyl group [20, 21]. Carboxylates interact with and stabilize calcium ions during precipitation of CaP [22]. The structure of the carboxymethyl hyaluronan (CMHA) used for these studies is shown in Figure 1.

It has been shown in our lab and others that stabilized nCaP can bind and release the chemotherapy drug* cis*-diamminedichloroplatinum (CDDP), a commonly used chemotherapeutic [23, 24]. CDDP is an effective anticancer drug but has dose limiting nephrotoxicity; thus, improved formulations with less toxicity are needed. We hypothesized that CMHA could be used to stabilize nCaP and simultaneously target CD44 expressing therapy resistant cells while delivering CDDP. The nCaP^CMHA^CDDP was physically characterized using transmission electron microscopy (TEM), X-ray diffraction, particle size analysis, and* in vitro *drug release studies. The ability of CMHA and nCaP^CMHA^CDDP to bind CD44 was examined using surface plasmon resonance. Cellular uptake was assessed using the CD44^+^ BT-474_EMT_ cells. Cytotoxicity of nCaP^CMHA^CDDP and the impact of CMHA on cytotoxicity of Aq CDDP were examined* in vitro *against both CD44^−^ BT-474 and CD44^+^ BT-474_EMT_ cell types. Lastly, an* in vivo* antitumor efficacy study was performedin a model of human therapy resistant TNBC using BT-474_EMT_ cells to create tumors in immunodeficient mice.

## 2. Materials and Methods

### 2.1. Materials

Calcium lactate pentahydrate (Sigma C8356), K_2_HPO_4_ (Sigma S1804), Pt(NH_3_)Cl_2_ (CDDP, Sigma P4394), and AgNO_3_ (Silver Nitrate, Sigma S6506) used to prepare the nanoparticles were all purchased from Sigma-Aldrich, (St. Louis, MO). Darvan 811 was purchased from R. T. Vanderbilt Holding Company, Inc. (Norwalk, CT). CMHA and HA for these studies were prepared as previously described [20, 21]. Aquated CDDP (Aq CDDP) that does not contain chloride ions is a net positive charged molecule that can bind to calcium phosphate nanoparticles and was prepared as described previously [23]. As a control, Aq CDDP was reacted with a solution of CMHA overnight to form an electrostatically bound conjugate and used to investigate the cytotoxicity of CDDP and CMHA without the calcium phosphate phase present.

BT-474 [25, 26] and BT-474_EMT_ cells were used for the cytotoxicity testing and* in vivo* tumor studies. BT-474_EMT_ were derived from human HER2-amplified epithelial BT-474 through serial mammosphere cultures over three weeks as previously described [27]. Serial mammosphere culture caused the BT-474 cells to undergo EMT and generated cells with a mesenchymal phenotype including enhanced proliferation rate, and cell surface marker expression of CD44^+^/CD24^−^ [28]. BT-474 cells were maintained in DMEM/F12 (Gibco 11330) with 10% FBS, 1% penicillin/streptomycin 10,000 U/mL (Gibco 15140), and 1% insulin (Gibco 41400). BT-474_EMT_ cells were maintained in DMEM/F12 (Gibco 11330) with 10% FBS, 1% penicillin/streptomycin 10,000 U/mL (Gibco 15140). BD Matrigel for cell injections was purchased from BD Biosciences (San Jose, CA).

J:Nu female mice were purchased from Jackson Laboratory (Bar Harbor, ME) and used for the* in vivo* tumor studies at 6–8 weeks of age. Female athymic nude mice, 6–8 weeks of age, were obtained from Charles River Laboratories International, Inc. (Wilmington, MA).

### 2.2. nCaP^CMHA^CDDP Production and Physical Characterization

Synthesis of nCaP^CMHA^CDDP was based on a previously reported method [23]. To make nCaP^CMHA^, an equal volume of 30 mM K_2_HPO_4_ was added to stirred 30 mM calcium lactate and immediately followed by addition of 2% (w/v) CMHA (34 kDa) in water at 20% of the total volume of precipitation. Nanoparticles were collected after 10 min of mixing via centrifugation (12000 rpm for 45 min) and washed once with MilliQ water. The nCaP^CMHA^ was resuspended at a concentration of 4 mg particles per mL of binding solution made of 1 : 1 (v : v) 20 mM potassium phosphate buffer and 1 mg/mL Aq CDDP. After 20 hours of binding protected from light on a heated rocker (LAB-LINE thermorocker, Barnstead Thermolyne, Dubuque, IA) maintained at 37°C, the particles were collected via centrifugation, rinsed with 10 mM KPB, and diluted to approximately 175 mg nCaP^CMHA^CDDP/mL MilliQ water. The suspension was injectable through a 25 gauge needle. All solutions/liquids during the synthesis process were sterile-filtered with a 0.2 *μ*m filter in order to prepare a sterile product for cell culture and animal testing. The nCaP^CMHA^CDDP suspension was stored at room temperature and shielded from light prior to use. Another type of nCaP stabilized with Darvan (nCaP^D^CDDP) instead of CMHA was made for use as a control for bulk shift during surface plasmon resonance studies. nCaP^D^CDDP was prepared as previously described [23] except that calcium lactate pentahydrate was used instead of Ca(NO_3_)_2_·4H_2_O, to match the calcium used in nCaP^CMHA^CDDP.

The concentration of CDDP within the nCaP^CMHA^CDDP and nCaP^D^CDDP was determined by inductively coupled plasma-optical emission spectroscopy (ICP-OES) (Perkin Elmer Optima 5300 DV, ESIS Inc., Cromwell, CT) after dissolution of the particles in 1 N HCl. Particle size analysis (PSA) was performed via dynamic light scattering using a 90 Plus Particle Sizer (Brookhaven Instruments, NY). Samples were prepared by sonicating the particle suspension and diluting the suspension 12x in MilliQ water. The morphology and size of the particles were observed by using a Hitachi H-7650 transmission electron microscope (TEM). TEM samples were prepared by sonicating the particle suspension and diluting the suspension 26x in MilliQ water and then 10x in 70% ethanol prior to placing a 5 *μ*L sample on a formvar carbon coated 300 mesh Cu grid and blotting excess solution. Prior to imaging the sample was completely dried in air for 5 min. Samples were imaged at 80 kV with the TEM. X-ray diffraction (XRD) was used to determine changes in crystallinity with addition of stabilizer and to compare dry versus wet nCaP. Samples of lyophilized calcium phosphate without stabilizer (microCaP), lyophilized nCaP^CMHA^, and wet nCaP^CMHA^ were analyzed using a Bruker D2 Phaser.

### 2.3. nCaP^CMHA^CDDP* In Vitro* Drug Release

*In vitro* drug release studies were performed using a USP apparatus 4 (Sotax CE, Sotax, Horsham, PA) modified with a dialysis adapter with a molecular weight cut-off of 100 kD [29]. A sample of 0.4 mL of particle suspension was loaded into the dialysis adapter with 100 *μ*L release medium. Release medium was 10 mM PBS pH 7.4 with 0.1% sodium azide. A flow rate of 8 mL/min was used for the study with the cell temperature maintained at 37°C. Release samples were drawn at 1 h, 3 h, 5 h, 7 hr, 12 hr, 1 d, 2 d, 3 d, 4 d, 5 d, 6 d, 7 d, and 10 d. At each time point 5 mL of release solution was taken and replaced with 5 mL of fresh PBS. CDDP content in the release solution was determined by ICP-OES. Media replacement during the release study was considered in the calculation of cumulative release.

### 2.4. Surface Plasmon Resonance

Interaction of nCaP^CMHA^CDDP, CMHA (~34 kDa), and HA (60 kDa) with CD44 were studied with surface plasmon resonance, BioRad ProteOn XPR36 with a GLC ProteOn sensor chip (BioRad, Hercules, CA). Recombinant human CD44-Fc chimera (~170 kDa) (R&D Systems, Minneapolis, MN) was immobilized on the sensor chip using amine coupling chemistry ProteOn Amine Coupling Kit. Briefly, the sensor chip surface was activated with 1 : 1 mixture of sulfo-N-hydroxysuccinimide (sulfo-NHS) and ethyl-3(3-dimethylamino)propyl carbodiimide (EDC) for 7 min. CD44-Fc was dissolved in 10 mM acetate buffer pH 4 to a concentration of 5 *μ*g/mL and flown over the activated surface for 14 min. The remaining reactive groups were blocked with 1 M ethanolamine HCl pH 8.5. A blank flow channel (FC) was prepared by EDC/NHS activation without the CD44 receptor. Throughout all the SPR measurements PBS pH 7.4 supplemented with 0.005% Tween 20 was used as the running buffer. Samples were diluted with running buffer. CMHA and HA were diluted to concentrations of 1 *μ*M and 5 *μ*M. nCaP^X^CDDP samples were diluted to a concentration of 350 *μ*g/mL, which was found to be a good compromise between sufficient binding response and bulk refractive index shift. The samples were injected over the sensor chip surface coated with human CD44-Fc at 100 *μ*L/min for 150 s. The dissociation in the running buffer took place for another 600 s. After each measurement cycle the sensor chip surface was regenerated with 10 mM glycine HCl pH 2.0 at a flow rate of 200 *μ*L/min. The responses on the blank flow cell were subtracted from the CD44-Fc coated flow cell.

### 2.5. Flow Cytometry

BT-474 and BT-474_EMT_ cells were analyzed for CD44 and CD24 expression using flow cytometry. Cells were washed once with phosphate-buffered saline (PBS) and then harvested with 0.05% trypsin/0.025% EDTA. Detached cells were washed with PBS that was supplemented with 0.2% (w/v) bovine serum albumin (BSA) (wash buffer) and resuspended in the wash buffer (10^6^ cells/100 *μ*L). Combinations of fluorochrome-conjugated monoclonal antibodies obtained from BioLegend (San Diego, CA) reactive against human and mouse CD44 (Alexa Fluor 647) and BD Pharmingen (San Jose, CA) CD24 (PE-Cy7) or their respective isotype controls were added to the cell suspension at concentrations recommended by the manufacturer and incubated at 4°C in the dark for 30 to 40 min. The labeled cells were washed in the wash buffer and then analyzed on a MACSQuant Analyzer, MACS Miltenyi Biotec (Auburn, CA).

### 2.6. Cellular Uptake Studies

Fluorescently labeled nCaP^CMHA-AF488^ was made via the method described in Section 2.2, with minor modifications. Briefly, Alexa Fluor 488 labeled CMHA was incorporated into the CMHA solution at 6% the total volume of CMHA used in the precipitation. All other reaction steps to create nCaP^CMHA^ were performed the same way. BT-474_EMT_ cells were seeded at a density of 1 × 10^5^ cells in an 8-well glass bottom plate and allowed to adhere for 24 hours after which nCaP^CMHA-AF488^ was added at the following concentrations: 200 *μ*g/mL, 1 mg/mL, or 2 mg/mL in 500 *μ*L complete media. After 2, 8, and 18 h after incubation, the glass slide chambers were washed 2x with PBS to remove any loose nanoparticles, and the cells were fixed 4% paraformaldehyde in PBS for 15 min. The fixed cells were washed with PBS 3x to remove the excess paraformaldehyde and then dried for 3 h. The fixed cells were stained and mounted with Prolong Gold antifade mounting media containing the nuclear stain 4′,6-diamidino-2-phenylindole (DAPI) (Thermo Fisher Scientific, Waltham, MA). Microscopic analysis was performed using a Nikon A1R Spectral Confocal Microscope. Conditions of the confocal microscopic analysis were a Z-stack thickness of 11 *μ*m, individual stack thickness of 0.35 *μ*m, and an oil immersed 40x objective.

### 2.7. Cytotoxicity

Cytotoxicity experiments were conducted using BT-474 (CD44 positive) and BT-474_EMT_ (CD44 negative) cells plated in 96-well plates at 6 × 10^4^ and 2 × 10^5^ cells/mL, respectively, with 50 *μ*L suspension per well. Two different seeding densities were used due to the very different growth rates of the cells during the cytotoxicity test. These cell types were selected to allow for the determination of cytotoxicity of nCaP^CMHA^CDDP relative to CD44 expression and to elucidate if CMHA enhances cell uptake and consequently cytotoxicity. BT-474 cells are CD44 negative and were thus used as a negative control. Cells were allowed to proliferate for 24 hours and then the test samples were added in an additional 50 *μ*L volume for a total of 100 *μ*L per well. The following groups were examined: CDDP, Aq-CDDP, and CMHA reacted with Aq CDDP (Aq CDDP-CMHA), nCaP^CMHA^CDDP, nCaP^CMHA^, and CMHA. Each group was serially diluted 1 : 3 across the plate using PBS. Cells were assayed 48 h after drug addition using in an MTS assay (CellTiter 96 AQ_ueous_ One, G3580, Promega Corp., Madison, WI), where metabolic activity was determined using a Spectramax Plus^384^ Spectrophotometer (Molecular Biosciences, Sunnyvale, CA) at an absorbance of 490 nm. To determine the IC50 (50% inhibitory concentration) a nonlinear regression curve fit analysis was performed with at least four replicates per group per concentration. All experiments shown were repeated two to three times.

### 2.8. BT-474_EMT_ Tumor Take Rate

To assess the growth parameters of BT-474_EMT_ tumors a tumor take rate study was performed in eight 6–8-week-old, immunocompromised athymic nude mice. Mice were given subcutaneous injections in the right rear flank via a 25-gauge needle of 5 × 10^5^ cells in a 100 *μ*L volume of BD Matrigel and base media (ratio of 60 : 40) based on the cell number utilized for a comparable transformed breast cancer cell type [30]. Animals were monitored at least every other day for normal grooming and appearance. Tumors were measured beginning at day 7 following inoculation. At this time point the Matrigel carrier has degraded allowing for a true cell-based tumor volume measurement.

### 2.9. nCaP^CMHA^CDDP* In Vivo* Maximum Tolerable Dose Determination

Two J:Nu immunocompromised mice with established BT-474_EMT_ tumors were injected once intratumorally with 10 mg/kg nCaP^CMHA^CDDP (80–90 *μ*L per injection). A second study was conducted using athymic nude mice (*N* = 4), where a 7 mg/kg dose of nCaP^CMHA^CDDP (60–70 *μ*L) was administered once intratumorally. For both studies animals were monitored daily for weight loss and grooming and euthanized if weight loss met or exceeded 15%.

### 2.10. nCaP^CMHA^CDDP* In Vivo* Antitumor Efficacy and Toxicity Study

An efficacy and toxicity study was performed using BT-474_EMT_ cells in J:Nu mice. The study included 24 6-week-old mice inoculated with 5 × 10^5^ BT-474_EMT_ cells in 100 *μ*L of a 60 : 40 ratio of BD Matrigel : cells in base media in the right rear flank via a 25-gauge needle. Tumors were measured daily 7 days following inoculation using digital calipers to calculate the tumor volume assuming an ellipsoid shape: *V* = (*W*)^2^ *∗L∗*0.4. Tumors were treated once with 2.8 mg/kg (60 *μ*L) CDDP NT (8 mice), 60 *μ*L of saline NT (4 mice), 60 *μ*L of nCaP^CMHA^ NT (4 mice), or 7 mg/kg nCaP^CMHA^CDDP NT (8 mice), when tumor volume reached 100 ± 10 mm^3^.

Systemic toxicity was evaluated by weight change and overall grooming/appearance. Tumor volume and mouse weight were monitored at least every other day. Mice were euthanized due to significant weight loss (>15%), a tumor length measurement greater than 2 cm, or completion of the study (day 30). At this time tumors were resected and weighed. All animal experimental procedures were approved by the Animal Care and Use Committee of the University of Connecticut Health Center (Farmington, CT).

### 2.11. Statistical Analysis

Statistical analysis was performed using an unpaired *t*-test (comparing two groups) or Tukey one-way ANOVA (comparing three or more test groups to a control group), as indicated in the methods. A *P* value of less than 0.05 was considered statistically significant. Data is presented as a mean value with its standard deviation indicated (mean ± SD).

## 3. Results and Discussion

**Figure 2:**
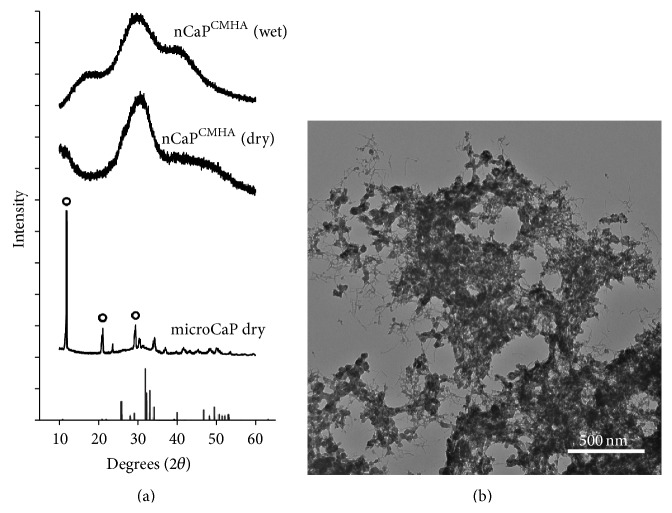
Physical characterization of nCaP^CMHA^ using X-ray diffraction and transmission electron microscopy. (a) XRD spectra of nCaP^CMHA^ suspension (nCaP^CMHA^ wet), lyophilized nCaP^CMHA^ (nCaP^CMHA^ dry), and lyophilized CaP without stabilizer added during precipitation (microCaP dry). Hydroxyapatite standard (JCPDS, #09-0432, bars) is shown for comparison. nCaP^CMHA^ both wet and dry has a major broad peak corresponding to the major peaks of hydroxyapatite. MicroCaP pattern has major peaks characteristic of brushite (peaks denoted by open circles). MicroCaP was precipitated without a stabilizer and has larger crystalline particles (narrow peaks). With the CMHA stabilizer present the crystallization is halted, depicted by broad peaks with little long range order. (b) TEM image of nCaP^CMHA^CDDP showing particles 20–50 nm in diameter clustered into larger, 200 nm, particles due to drying prior to imaging.

The physical characteristics of nCaP^CMHA^CDDP are shown in Table 1. Precipitation of nCaP^CMHA^ resulted in an efficient yield of 2.3 ± 0.4 mg per mL of precipitation solution. The CDDP concentration in the nCaP^CMHA^CDDP suspension was 4.1 ± 1.4 mg/mL, with a drug loading of 140 ± 12 *μ*g CDDP/mg nCaP^CMHA^. nCaP^CMHA^CDDP were on average 204 ± 13 nm in diameter as measured by dynamic light scattering with a polydispersity of 0.116. The zeta potential of the particles was −43 ± 4 mV, which is greater than the colloidal stability threshold of ±30 mV thereby providing evidence of enhanced stability due to surface charge prevention of aggregation [31]. XRD data showed nCaP^CMHA^ was poorly crystalline apatite based on the major peak occurring at 30° which corresponds well with hydroxyapatite. MicroCaP made without the CMHA stabilizer was more crystalline in nature resembling brushite and poorly crystalline hydroxyapatite (Figure 2(a)) [32, 33]. The introduction of CMHA clearly restricts the crystallization of CaP, as can be seen by the broad peaks of nCaP^CMHA^ XRD spectra relative to the spectra of microCaP which exhibited a crystalline pattern with major peaks of hydroxyapatite as well as brushite (calcium hydrogen phosphate dehydrate, CaHPO_4_·2H_2_O). Transmission electron microscopy (TEM) (Figure 2(b)) revealed that nCaP^CMHA^CDDP in suspension forms small aggregates that correlate well with their measured particle size using DLS, 204 ± 13 nm. The addition of CMHA during precipitation of CaP resulted in successful stabilization of nCaP. TEM images revealed small 30–80 nm particles, agglomerated into larger particles, which likely accounts for the 200 nm size measured by DLS. These characteristics of the nCaP^CMHA^CDDP resulted in a clog-free injectable nanoparticle suspension via a 25G needle.

**Figure 3:**
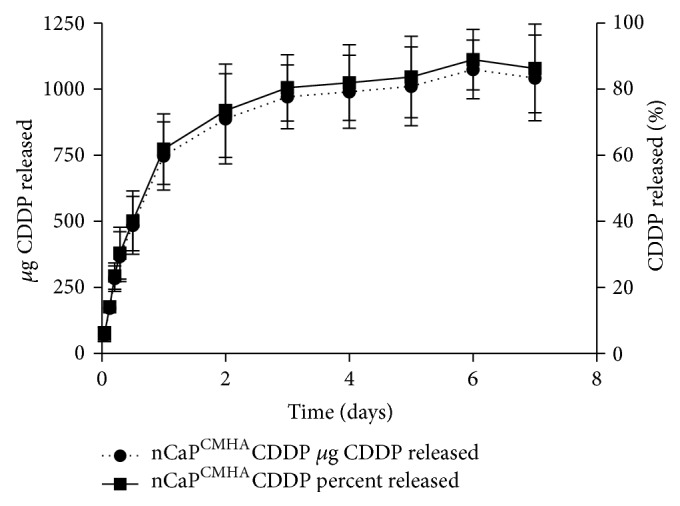
*In vitro *release testing of nCaP^CMHA^CDDP using a modified USP Apparatus 4. Release was conducted in 10 mM PBS pH 7.4, 0.1% sodium azide at 37°C. The left axis is cumulative CDDP released with percent CDDP released on the right *y*-axis. The formulation provides sustained delivery of CDDP for 2 days under these infinite sink conditions. After 2 days nCaP^CMHA^CDDP released 74% of the total CDDP bound. At day 7, nCaP^CMHA^CDDP released an average of 86% of the total CDDP bound.

To design an effective nanoparticle drug delivery system it is essential to optimize the physicochemical interactions of each component: the carrier, the drug, and any biological targeting moiety [34]. It was shown in previous work that sodium polyacrylate (Darvan 811, D) halts CaP crystal growth [23]. This is due to the repeating carboxylate groups throughout the polymer. There is significant literature showing the carboxylate groups of sodium citrate (3 carboxylate groups per molecule) interact with the Ca ions during CaP precipitation acting as a surfactant to halt nucleation [22, 35–38]. Though these molecules effectively stabilize CaP, they have no biological targeting capacity. We therefore used a stabilizer that has biological targeting capability concurrent with nCaP stabilization. CMHA is a modified HA with additional carboxylate groups, thus allowing for more effective nCaP stabilization. CMHA at a degree of modification below 25% binds to HA binding proteins with comparable affinity and avidity as native HA. Importantly, cross-linked hydrogels based on thiolated CMHA improve wound healing in cutaneous and ophthalmic injuries [39], allow delivery of small molecules as well as growth factors and other macromolecules [40], and are safe and effective vehicles for cell delivery and retention [40]. Clinical products based on CMHA are in development for cell therapy [41]. Thus, CMHA was selected to enhance uptake of nCaP^CMHA^CDDP by cells expressing CD44. After confirming that a stable calcium phosphate nanoparticle was achieved, we tested whether these nanoparticles could bind, release, and maintain biological efficacy of CDDP. The* in vitro *release of the nCaP^CMHA^CDDP in PBS, pH 7.4, at 37°C over time can be seen in Figure 3. Under the rigorous, high speed and high volume release conditions nCaP^CMHA^CDDP exhibited a burst release of 73% of the CDDP bound in two days which plateaued and reached a total of 86% after seven days.

**Figure 4:**
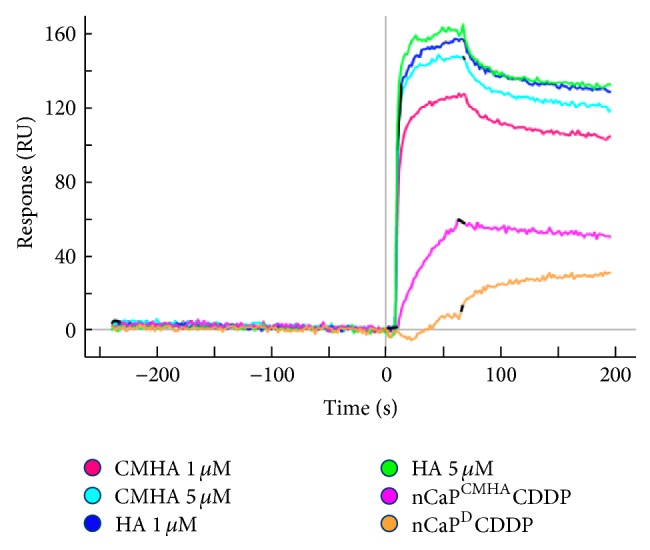
Surface plasmon resonance sensogram depicting binding of CMHA, HA, and nCaP^CMHA^CDDP with immobilized CD44. All data shown has been corrected for nonspecific binding to blank channels of blocked NHS-EDC. nCaP^D^CDDP was used as a comparable sized control, which does not have specific interactions with CD44. HA has the highest affinity for CD44, followed by CMHA then nCaP^CMHA^CDDP.

Surface plasmon resonance (SPR) was performed to assess the interaction and binding of CMHA, nCaP^CMHA^CDDP, and HA to CD44. Human CD44 chimera was immobilized on four channels of the sensor chip and two channels were amine coupled and blocked serving as a negative control for nonspecific binding. To correct for bulk shift due to the size of the nanoparticles, nCaP^D^CDDP was examined as a control. As expected, HA most effectively bound to CD44 with CMHA approaching, but not equaling, the binding affinity of HA (Figure 4). nCaP^CMHA^CDDP also binds CD44, but this binding is lower than free CMHA or HA. Importantly, this binding is specific as it overcomes any bulk interactions observed with nCaP^D^CDDP. SPR analysis of targeted nanoparticles is challenging. SPR systems utilize complex microfluidics that normally transport solutions containing ligands or proteins of interest, but generally not solid materials such as nCaP. Of additional concern is the ability to correct for bulk shift due to the relatively large nanoparticles passing over the sensor. nCaP^D^CDDP served as a comparably sized, nonspecific nanoparticle control. The density of the receptor (CD44) immobilized on the chip is inherently related to the response measured; therefore, we utilized a low density of CD44 on the chip surface [42]. The highest binding observed was for HA (60 kDa), followed by CMHA (34 kDa). The chemical modification of HA to create CMHA occurs at 15–20% of the repeating 6′-OH groups of the N-acetylglucosamine residues. The interaction of CD44 and HA has low affinity but high avidity. A single HA disaccharide contains an N-acetyl-D-glucosamine and D-glucuronic acid, HA_2_. It has been shown that HA_6_ is necessary for binding CD44, but HA_10_ is preferred [43]. Additionally, divalent binding occurs with HA_20_ and larger oligomers. This likely explains the slight reduction in binding of CMHA to CD44, due to an interruption of sugar residues by the added carboxylate groups of CMHA compared to HA.

**Figure 5:**
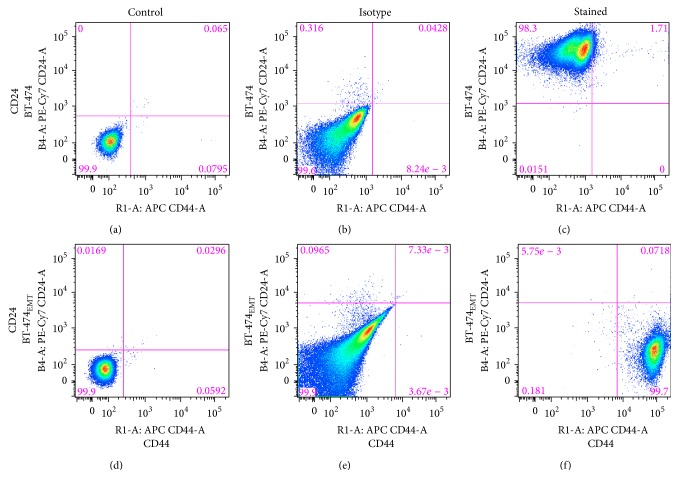
Flow cytometry data for the BT-474 and BT-474_EMT_ cells demonstrates appropriate CD44 expression for the studies. The numbers in the corner of each quadrant are the percentage of positive cells within the quadrant. (a) BT-474 unstained control, (b) BT-474 isotype control, (c) BT-474 stained cells with CD44, Alexa Fluor 647, and CD24, PE-Cy7. BT-474 cells are CD44^low^/CD24^high^. These cells served as the negative control for CD44 targeting, because they lack CD44 expression. (d) BT-474_EMT_ unstained control, (e) BT-474_EMT_ isotype control, (f) BT-474_EMT_ stained cells with CD44, Alexa Fluor 647, and CD24, PE-Cy7. BT-474_EMT_ cells are CD44^+^/CD24^low^. These cells were tested to investigate CD44 targeting, as they have high CD44 expression.

**Figure 6:**
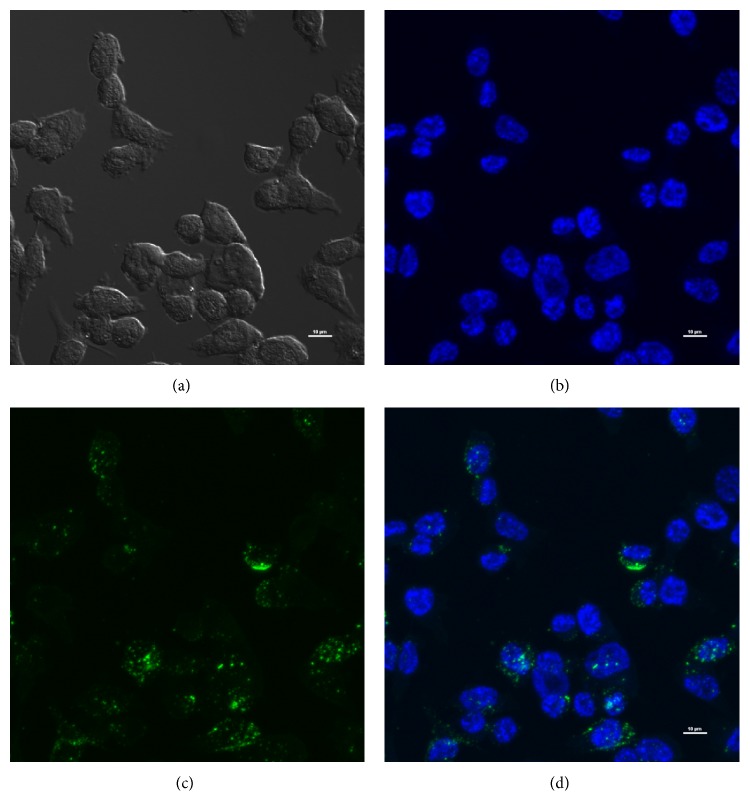
Cellular uptake study using BT-474_EMT_ cells. Cells were plated and allowed to adhere for 24 hours, after which nCaP^CMHA-AF488^ was added at a concentration of 2 mg/mL. After 18 hours, nCaP^CMHA-AF488^ can clearly be seen within cells as confirmed by Z-stack confocal images. (a) Differential interference contrast (DIC) image, (b) cells stained with DAPI, (c) cells imaged containing nCaP^CMHA-AF488^, and (d) overlay of DAPI and AF488 images, showing nCaP^CMHA-AF488^ uptake.

Flow cytometry analysis confirmed BT-474 are 1.71% positive for both CD44 and CD24 and the majority of cells stained positively for CD24 alone, 98.3% (Figures 5(a)–5(c)). These cells were thus a good negative control for CD44 targeting. BT-474_EMT_ cells stained 99.7% positive for CD44 and negative for CD24, which corresponds well to the phenotype of therapy resistant breast cancer cells in the literature, CD44^+^/CD24^−/low^ (Figures 5(d)–5(f)) [11, 44]. BT-474_EMT_ cells were therefore used as the CD44^+^ test groups of cells to examine CD44 specificity with cellular uptake and cytotoxicity of nCaP^CMHA-AF488^. In the cell uptake studies no significant uptake was determined for the 200 *μ*g/mL or 1 mg/mL concentrations at any time tested (images not shown). At the 2 mg/mL dose, significant cellular uptake was determined at 18 hours posttreatment (Figures 6(a)–6(d)). Z-stack images were obtained, confirming the nCaP^CMHA-AF488^ was within the cell with nuclei counterstained with DAPI. This was a preliminary test of cellular uptake. These were insufficient to prove that nCaP^CMHA-AF488^ cellular uptake was mediated by CD44. To do so, at least two controls are necessary, a CD44^−^ cell type and a CD44^+^ cell type pretreated with HA to saturate the CD44 receptors [45] and should be included in future studies. SPR showed nCaP^CMHA^CDDP had lower binding than CMHA, which is likely due to two factors. Only 30% of the 4 mg/mL CMHA in the precipitation is incorporated into nCaP^CMHA^. Additionally, nCaP^CMHA^CDDP is stored as a suspension which allows the CaP to undergo Ostwald ripening incorporating much of the CMHA within the nCaP core [46, 47]. The exposure time required for cellular uptake of nCaP^CMHA-AF488^ was more than four times that of mesoporous silica nanoparticles targeted to CD44 via HA, where a 200 kDa HA was used [15].

**Figure 7:**
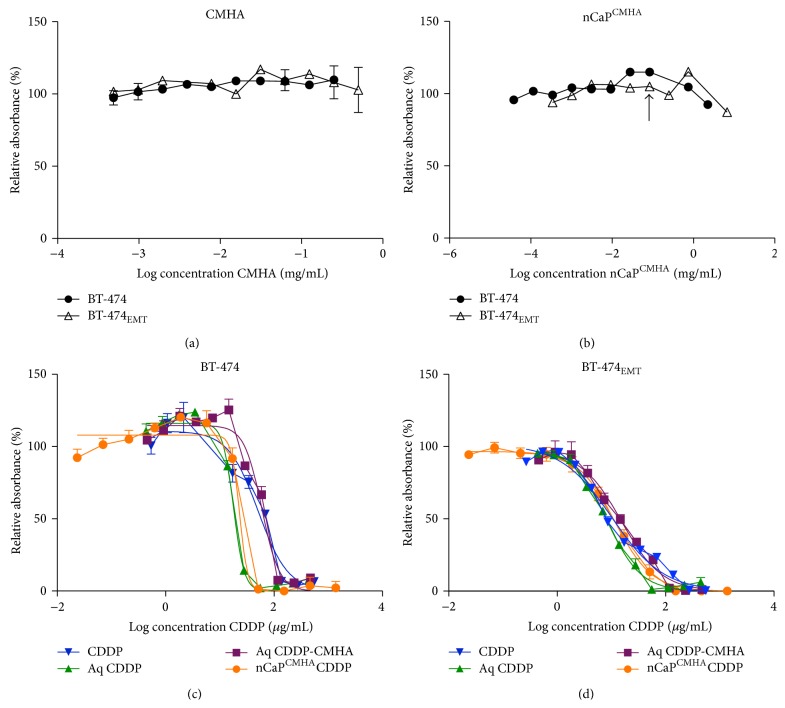
Cytotoxicity examination of CMHA, nCaP^CMHA^, CDDP, Aq CDDP, Aq CDDP-CMHA and nCaP^CMHA^CDDP evaluated against BT-474 and BT-474_EMT_ cells using an MTS assay. (a) Evaluation of CMHA alone against BT-474 and BT-474_EMT_ cells, showing no cytotoxicity. (b) Evaluation of nCaP^CMHA^ alone against BT-474 and BT-474_EMT_ cells, showing no cytotoxicity. (c) Cytotoxicity evaluation using BT-474 (CD44^−^) cells. CDDP, Aq CDDP, and Aq CDDP reacted with CMHA (Aq CDDP-CMHA), and nCaP^CMHA^CDDP curves are plotted. (d) Cytotoxicity evaluation using BT-474_EMT_ (CD44^+^) cells. CDDP, Aq CDDP, and Aq CDDP reacted with CMHA (Aq CDDP-CMHA), and nCaP^CMHA^CDDP curves are plotted.

The cytotoxicity of CMHA, nCaP^CMHA^, CDDP, Aq CDDP, Aq CDDP-CMHA, and nCaP^CMHA^CDDP was examined against BT-474 (CD44 negative) and BT-474_EMT_ (CD44 positive) cells. These studies confirmed that CMHA did not have any inherent toxicity to either cell type (Figure 7(a)). The CMHA stabilized nCaP^CMHA^ also had no cytotoxicity when tested at the concentrations which matched the amount of nCaP in the nCaP^CMHA^CDDP test groups (Figure 7(b)). The IC50 curves of CDDP, Aq CDDP, and Aq CDDP reacted with CMHA (Aq CDDP-CMHA), and nCaP^CMHA^CDDP against BT-474 cells are shown in Figure 7(c). The IC50 curves of CDDP, Aq CDDP, and Aq CDDP reacted with CMHA (Aq CDDP-CMHA), and nCaP^CMHA^CDDP against BT-474_EMT_ cells are shown in Figure 7(d). The IC50 values calculated from these curve fits are shown in Table 2. nCaP^CMHA^CDDP and Aq CDDP were significantly more cytotoxic than CDDP alone against BT-474 cells and Aq CDDP-CMHA was significantly less cytotoxic (*P* ≤ 0.001). nCaP^CMHA^CDDP had comparable cytotoxicity to CDDP against BT-474_EMT_ cells. Aq CDDP-CMHA was significantly less cytotoxic than CDDP (*P* ≤ 0.001) and Aq CDDP was significantly more cytotoxic (*P* ≤ 0.05) for BT-474_EMT_ cells.

Interestingly, nCaP^CMHA^CDDP did not show preferential cytotoxicity to cells with high CD44 expression (BT-474_EMT_) compared to those with negative CD44 expression (BT-474), or compared to CDDP alone. It was hypothesized nCaP^CMHA^CDDP would target cells with high CD44 expression causing increased cytotoxicity. In related studies, mesoporous silica nanoparticles targeted with HA and carrying doxorubicin were significantly more cytotoxic than doxorubicin alone against CD44 expressing cells but were significantly less cytotoxic to CD44 negative cells [16]. Similarly, an HA Taxol prodrug was more cytotoxic than free Taxol against cells expressing CD44 and had limited to no cytotoxicity against cells that did not express CD44 [13, 14]. These studies demonstrate a specific CD44 mediated uptake in the HA containing particles that allowed the drug to release intracellularly, where cells lacking CD44 did not take up the prodrug. Possible reasons for the opposite findings in our studies are as follows. While nCaP^CMHA^ was taken up by BT-474_EMT_ cells, the uptake was relatively slow compared to other HA nanoparticle formulations which were taken up after 4 hours of exposure [15, 16]. The SPR data implies that the HA presentation after adsorption to the CaP was not optimal. The future use of CMHA with greater spacing between the carboxylate groups may overcome this. Additionally it was expected that the BT-474_EMT_ cells would be chemotherapy resistant and would require a carrier to enhance effectiveness of CDDP, since comparable mesenchymal-derived MCF-7 cells were resistant to other chemotherapies (docetaxel and tamoxifen) [11, 30]. However, the cytotoxicity data shows that BT-474_EMT_ cells are sensitive to CDDP as compared to BT-474 cells without CD44. The major adducts caused by CDDP are 1,2-intrastrand cross-links that interfere with DNA replication and transcription resulting in cell death during G2 phase prior to mitosis [48]. Unexpectedly the nCaP^CMHA^CDDP was more cytotoxic than CDDP alone against the BT-474 cells without CD44. The controlled release of CDDP from nCaP^CMHA^CDDP allowed for prolonged delivery of drug to the slower replicating BT-474 cells, which could explain the enhanced cytotoxicity of nCaP^CMHA^CDDP over CDDP alone.

**Figure 8:**
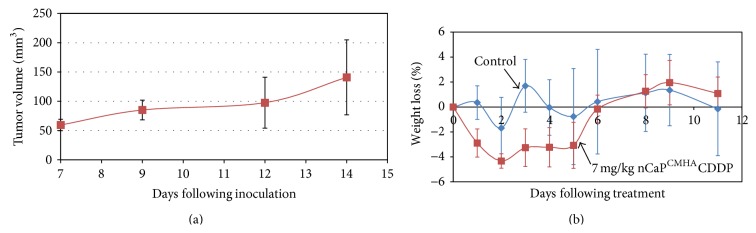
*In vivo *tumor take rate and maximum tolerable dose studies. (a) Tumor take rate study performed in athymic nude mice with 5 × 10^5^ BT-474_EMT_ cells injected subcutaneously in right rear flank of animals. Data represents average tumor volume versus days following inoculation with standard deviations. (b) Maximum tolerable dose study conducted with athymic nude mice (6–8 weeks old) carrying BT-474_EMT_ tumors. An intratumoral 7 mg/kg dose of nCaP^CMHA^CDDP (4 mice/group) was compared to an untreated control (4 mice/group). nCaP^CMHA^CDDP caused minimal weight loss at 7 mg/kg and all animals recovered.

Importantly, the nCaP^CMHA^CDDP had equivalent cytotoxicity to CDDP* in vitro *which means nCaP^CMHA^ had no negative impact on the biological activity of CDDP and thus these particles were suitable candidates for evaluation in an* in vivo* tumor model. The* in vivo* tumor forming capability of BT-474_EMT_ cells had not been previously studied; therefore, their ability to form tumors was first examined in a tumor take rate study. BT-474_EMT_ tumors formed after cell injections were 50 mm^3^ on average after 7 days. After approximately 12 days tumors were 100 mm^3^ (Figure 8(a)). Animals were monitored for 25 days following inoculation where tumors continued to grow steadily up to 500 mm^3^ without necrosis. The comparable mesenchymal-derived human MCF-7 human BC cells were also tested under the same conditions in an* in vivo* tumor model and were found to have similar growth rates as the BT-474_EMT_ with volumes reaching 500 ± 100 mm^3^ at 20 days following inoculation [30]. These studies confirmed that this new human breast cancer tumor model BT-474_EMT_ was not too fast growing and did not develop necrotic tumors.

A maximum tolerable dose (MTD) study was conducted in mice bearing BT-474_EMT_ tumors. Based on a small pilot study showing excessive weight loss in at least one mouse mice treated with 10 mg/kg nCaP^CMHA^CDDP, mice were given 7 mg/kg nCaP^CMHA^CDDP (60–70 *μ*L) intratumorally into tumors with an average tumor volume of 170 mm^3^, 14 days following cell inoculation. The results are shown in Figure 8(b), where the maximum weight loss was 5% occurring at two to five days following treatment. This is an acceptable weight loss during treatment with chemotherapeutics; therefore, this dose was deemed tolerable and used for the antitumor studies.

**Figure 9:**
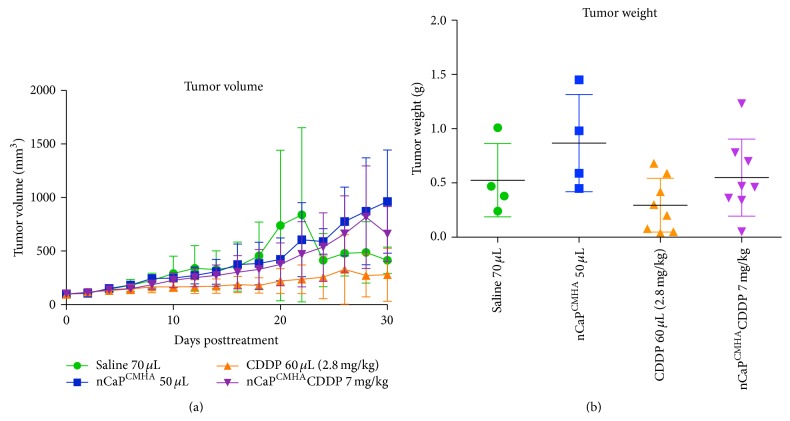
Efficacy study of nCaP^CMHA^CDDP conducted on J:Nu mice bearing BT-474_EMT_ tumors. Animals were treated once when their tumor volume reached 100 ± 10 mm^3^ and were compared to 2.8 mg/kg CDDP administered near the tumor. Tumor volume, grooming, and weight loss were monitored every other day following treatment. (a) The graph depicts average tumor volume (mm^3^) per group versus days posttreatment. The negative control saline IT (70 *μ*L) had no effect on tumor growth. nCaP^CMHA^ (60 *μ*L) had no effect on tumor growth. CDDP at 2.8 mg/kg administered near the tumor delayed tumor growth. (b) Tumor weight at the end of the study or at time of euthanasia for the efficacy study shown in (a). Tumors were resected and weighed. Animals were euthanized if tumor diameter was measured > 2 cm or at the completion of the study (day 30). No significant differences were found between groups.

**Figure 10:**
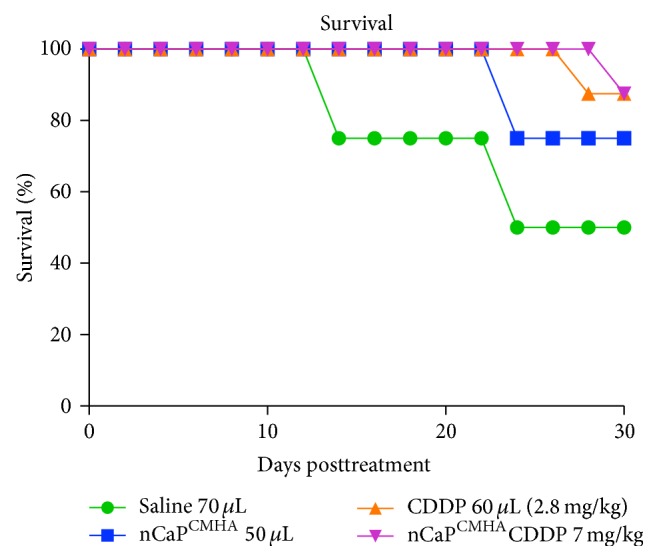
Survival was plotted for the efficacy study shown in Figure 9. The study was conducted on J:Nu mice bearing BT-474_EMT_ tumors. Animals were treated once when their tumor volume reached 100 ± 10 mm^3^ and compared to 2.8 mg/kg CDDP administered near tumor (NT). Tumor volume, grooming, and weight loss were monitored every other day following treatment. Survival was defined as a tumor diameter > 2 cm or inability to groom. A treatment of 7 mg/kg nCaP^CMHA^CDDP and 2.8 mg/kg CDDP (NT) was most effective at prolonging survival compared to control treatments of saline or nCaP^CMHA^ (NT).

To assess the antitumor efficacy of the nCaP^CMHA^CDDP, tumors were treated once by direct injection near the tumor with one of the following treatments: 2.8 mg/kg (60 *μ*L) CDDP (8 mice), 60 *μ*L of saline (4 mice), 60 *μ*L of nCaP^CMHA^ (4 mice), or 7 mg/kg nCaP^CMHA^CDDP (8 mice), when tumor volume reached 100 ± 10 mm^3^. No significant differences in tumor volume or weights were found among groups (Figure 9(a)), even the positive control (CDDP only). No animal experienced weight loss greater than 2% at these doses (data not shown). At the time of euthanasia, tumors were resected and weighed to compare to volumetric measurements. The resulting tumor weights were plotted relative to treatment (Figure 9(b)). No significant differences were found between groups when analyzed according to tumor weight. No treatment caused toxicity to the animals as measured by weight loss and overall grooming/appearance. Survival over time posttreatment was evaluated for each group (Figure 10). Mice were not actually allowed to die due to the treatments or tumor growth; instead they were euthanized if the tumors reached a tumor length measurement greater than 20 mm. In this slow growing model half of the untreated animals still had small tumors at the end point of the study making it difficult to observe large changes in tumor size due to treatment. Large standard deviations of tumor volume in the untreated group (as well as treatment group) were another problem obscuring differences between groups. In our studies with the FaDu (human head and neck squamous cell carcinoma) tumor model, it was necessary to use a larger number of cells (2 × 10^6^ cells) to achieve reproducible growth rather than 5 × 10^5^ cells (which was used in these studies with the BT-474_EMT_). If a higher number of BT-474_EMT_ cells were injected, limited variability in tumor volume might have occurred enabling a more discriminating evaluation of the test groups.

At the time of resection, depots of nCaP^CMHA^CDDP of a similar size to that injected were observed adjacent to the tumor on one or both sides indicating the nCaP was not resorbed or degraded over one month of implantation. Visual observations of the placement of the material allowed us to make a correlation between treatment placement and efficacy. The nCaP^CMHA^CDDP had the greatest antitumor effect when it was evenly distributed around the tumor. The lack of an even distribution nCaP^CMHA^CDDP may have contributed to the lack of antitumor efficacy observed in these animals. nCaP^CMHA^CDDP did not freely diffuse throughout the tumor due to the density of the tumor, and therefore only the areas of the tumor proximal to the nCaP^CMHA^CDDP depot were exposed to the CDDP. It is likely that the ability of free CDDP to diffuse throughout the tumor caused the more reproducible tumor growth inhibition observed in the CDDP alone group. Intravenous administration of the nanoparticles may allow for greater penetration of the treatment into the tumor interior with an associated increased antitumor effect. A recently published clinical trial shows that neoadjuvant CDDP intravenous treatment was effective for patients with TNBC, though this treatment has not yet been adopted clinically [49]. Therefore, continued investigations on CDDP delivery systems are warranted for TNBC.

## 4. Conclusions

CMHA is a novel and effective stabilizer for nCaP that can bind to CD44 receptors for potential targeting applications. Importantly, it was determined that CMHA has no negative impact on the biological activity of CDDP* in vitro*, against human breast cancer cells. nCaP^CMHA^CDDP allowed for efficient release of CDDP. nCaP^CMHA^CDDP had equivalent cytotoxicity to CDDP alone against CD44^+^ cells and was more cytotoxic for CD44^−^ cells. Tumors are very heterogeneous in cell surface expression and therefore cytotoxicity to both CD44^+^ and CD44^−^ cells is important* in vivo*. In future studies, an intravenous dose of nCaP^CMHA^CDDP at the maximum tolerable dose with optimized cell uptake and tumor infiltration should be examined* in vivo *to evaluate the targeting of therapy resistant CD44^+^ cells by chemically modified HA.

## Figures and Tables

**Table 1:** Average physical characteristics of nCaP^CMHA^CDDP. Ratio of components, precipitation volume, and stabilizer final concentration remained the same from batch to batch. Yield, CDDP concentration, drug loading, particle size, polydispersity, and zeta potential represent averages and standard deviations from a minimum of three batches.

Physical characteristic	nCaP^CMHA^CDDP
Ca : P : Stabilizer (v : v : v)	2 : 2 : 1
Total precipitation volume (mL)	250
Stabilizer final concentration (mg/mL)	4
Yield (mg nCaP/mL precipitation)	2.3 ± 0.4
CDDP concentration (mg/mL)	4.1 ± 1.4
Drug loading (*μ*g CDDP/mg nCaP)	140 ± 12
Particle size (nm)	204 ± 13 nm
Polydispersity	0.116
Zeta potential (mV)	−43 ± 4

**Table 2:** Tabulated IC50 values of CDDP, Aq CDDP, Aq CDDP-CMHA, and nCaP^CMHA^CDDP examined with BT-474 and BT-474_EMT_ cells, from curves shown in Figures 7(a) and 7(b), respectively. For BT-474 cells Aq CDDP and nCaP^CMHA^CDDP were both significantly more effective than CDDP alone and Aq CDDP-CMHA was significantly less effective than CDDP. For BT-474_EMT_ cells nCaP^CMHA^CDDP was as cytotoxic as CDDP alone and Aq CDDP was significantly more effective while Aq CDDP-CMHA was significantly less effective.

Cell type	IC50 (*μ*g/mL)			
	CDDP	Aq CDDP	Aq CDDP-CMHA	nCaP^CMHA^CDDP
BT-474	50.6 ± 3.3	17.1 ± 0.9^c^	59.0 ± 2.7^c^	23.1 ± 3.9^c^

BT-474_EMT _	10.5 ± 1.1	8.78 ± 0.6^a^	16.24 ± 1.5^c^	12.1 ± 1.4

^a^ *P* ≤ 0.05.

^c^ *P* ≤ 0.001.
